# Multistage carcinogenesis in occupational cholangiocarcinoma: the impact of clonal expansion and risk estimation

**DOI:** 10.1186/s41021-024-00315-7

**Published:** 2024-10-24

**Authors:** Masahiko Watanabe, Hiroshi Haeno, Sachiyo Mimaki, Katsuya Tsuchihara

**Affiliations:** 1 School of Pharmacy, Shujitsu University, 1-6-1 Nishigawara, Okayama, 703–8516 Japan; 2 Research Institute for Biomedical Sciences, Tokyo University of Science, 2669 Yamazaki, Noda, Chiba, 277–0022 Japan; 3 Division of Translational Informatics, Exploratory Oncology Research and Clinical Trial Center, National Cancer Center, 6-5-1 Kashiwanoha, Kashiwa, Chiba, 277–8577 Japan; 4 Exploratory Oncology Research and Clinical Trial Center, National Cancer Center, 6-5-1 Kashiwanoha, Kashiwa, Chiba, 277–8577 Japan

**Keywords:** Occupational cholangiocarcinoma, Intrahepatic cholangiocarcinoma, Multistage model, Mutation, Clonal expansion, Risk estimation

## Abstract

**Background:**

Both mutation induction and clonal expansion of mutated cells cause cancer. The probability of cancer development depends on mutations, clonal growth rates, and carcinogenic mechanisms. A recent study showed cases of occupational cholangiocarcinomas that originate multifocally, with higher mutation burden levels than those in common cholangiocarcinomas. This study aimed to identify the effect of clonal expansion on and estimate the risk of occupational and common intrahepatic cholangiocarcinomas (ICCs) using a multistage model modified to include the effect of cell expansion at any carcinogenic stage.

**Methods:**

The age-specific incidence of common ICC estimated from the Vital Statistics in Japan and the prognosis of ICC, and mutation frequencies of occupational and common ICC available from the previous report, were applied to a multistage model modified with cell proliferation effects. From the fittest model, the risk after exposure was estimated.

**Results:**

The required number of stages for carcinogenesis was estimated to be three based on the incidences and mutation frequencies of occupational and common ICCs. Based on this estimation, the predicted incidence curve under the model was similar to that estimated from the ICC mortality rate, except for older adults. The model indicated a minor effect of clonal expansion on the observed occupational ICC risk. It predicted a rapid decrease in ICC risk after the cessation of occupational exposure, although the time of clinical detection of cancer after the exposure was affected by latency. The model predicted an increase in cancer risk in older adults caused by cell expansion and common background mutations. However, the risk in older adults was overestimated in the case of common ICC; this divergence could influence occupational ICC cases.

**Conclusions:**

Three-stage ICC carcinogenesis has been proposed. The high mutation burden levels caused by occupational exposure led to an immediate incidence of cancer. After a long period of relatively low cancer risk, an increased risk in older adults was also predicted.

**Supplementary Information:**

The online version contains supplementary material available at 10.1186/s41021-024-00315-7.

## Introduction

The accumulation of somatic mutations drives carcinogenesis in cells. Passenger mutations account for most somatic mutations in cancer cells and are not subject to selective pressure during carcinogenesis. Driver mutations that occur during carcinogenesis have various effects on mutant cells, such as proliferation acceleration and genetic and chromosomal instabilities. These effects increase cancer incidence and affect the mutation frequency of the resulting cancer cells. Mutated cell expansion results from a selective growth process of cells that have driver mutations and causes further mutations during cell division.

Various cancer models have explained the age-dependent cancer incidence rates. Armitage and Doll’s multistage model is a classic that mathematically expresses the exponential increase in cancer mortality by age [1]. This model presumes that stage progression occurs at a constant rate. The cumulative incidence or mortality is then calculated to be proportional to the *k*-th power of age on the *k*-stage model. As premalignant cell expansion promotes carcinogenesis, the two-stage clonal expansion model has also been proposed [2, 3]. In this model, the first stage is initiation, followed by clonal expansion and malignant transformation in the second stage. This model has been extended to the multistage clonal expansion model [4, 5], which presumes more than one step to reach initiation and acquire the clonal growth ability. Various cancer models have been proposed, including the integration of variable factors such as genome instability and selective forces [6–8] and in cases of recurrence [9, 10].

A decade ago, the incidence of occupational cholangiocarcinomas caused by occupational exposure to 1,2-dichloropropane and dichloromethane was reported [11]. The somatic mutation frequency in occupational cholangiocarcinomas was significantly higher than that of common cholangiocarcinomas [12]. Exposure to extremely high concentrations of organic solvents could cause multiple mutations in many driver genes to occur in a cell, which may lead to carcinogenesis even without the subsequent long-term processes, including the clonal expansion of the mutated cells and further mutation accumulation in driver genes.

The outbreak of these occupational cholangiocarcinomas is of great concern; hence, investigating the carcinogenic mechanisms and assessing incident risks are necessary. Meanwhile, the integrated modeling and computation of a wide variety of carcinogenic processes requires a high level of numerical handling, and estimating the various parameters is usually difficult. Whole exome sequencing analysis revealed that several patients with cholangiocarcinomas harbored multiple cancerous lesions, of which some arose independently [13]. This study showed a mathematical model of multistage carcinogenesis, including cell expansion, and applied the somatic mutation data of normal and occupational intrahepatic cholangiocarcinomas (ICC) were applied to the model. The mechanism of carcinogenesis in occupational cholangiocarcinomas proposed from the mathematical model based on the statistical data and future risk estimation were discussed.

## Methods

### ICC incidence

Data from the Vital Statistics in Japan were used in this study [14]. As almost all cases of occupational cholangiocarcinoma occurred in males, they were the focus of this study. The mortality rate of every five-year age group was calculated from the statistical data of five-year age groups, including the total population and number of deaths by ICC (Table S1). Mortality rates of males caused by ICC by age in 1999 and 2019 are shown in Fig. S1. Until age 85, the mortality rate curves showed a similar pattern between 1999 and 2019. This study utilized the average mortality rate from 2015 to 2019 to reduce variations among sampling years. To determine the cumulative risk, cumulative mortality was calculated by integrating the mortality rates of the age groups. As the ICC incidence rate was not generally available, it was estimated from the survival rate and presumed lag time. The 10-year relative survival rates [15] of liver cancer and gallbladder/bile duct cancer in males diagnosed in 2002–2006 were 9.6% and 18.5%, respectively. During 1997–1999, their five-year survival rates were 23.7% and 21.8% [16]. Recent improvements in cancer treatments caused increased five-year survival rates to be 36.2% and 26.8%, respectively, for those diagnosed during 2009–2011 [17]. Although large ICC cohort datasets were not available to determine the survival rates, three- and five-year survival rates of ICC with surgical resection from 2005–2015 in National Cancer Center Hospital East were 47% and 37%, respectively [18], which indicates that ICC is also a relatively intractable cancer. A complete cure is uncommon, and most patients will die after several years. For occupational cholangiocarcinomas, the eight-year survival rate after resection was approximately 40%, though 15 of the 37 patients could not be surgically resected [19]. Another study demonstrated that young patients with ICC mostly died within five years following diagnosis, and the five-year survival rate was 23.1% [20]. Based on these data, the overall survival rate was assumed to be 20%, and the lag time between the malignant transformation in the model and the fatal event in the model was assumed to be five years. From this assumption, the cumulative incidence of ICC was calculated (Table S1).

### Assuming the stem cell number for ICC

Hepatic stem cells are multipotent and can differentiate into hepatocytes and cholangiocytes [21, 22]. Therefore, the cell number was estimated based on the relative risk of ICC to liver cancer per stem cell and the incidence rates of ICC and liver cancer. The number of the stem cells was 3.01 × 10^9^ [23]. Several risk factors of liver cancer affect the relative risk of ICC and liver cancer, e.g., hepatobiliary flukes increase the incidence of cholangiocarcinoma more than that of hepatocellular carcinoma; in contrast, hepatitis C virus increases the incidence of hepatocellular carcinoma more [24–26]. In Japan, the proportion of ICC mortality risk in liver cancer rose from 5% in 1999 to more than 15% in 2019, probably because of the lower contribution of the hepatitis virus. The number of stem cells responsible for ICC was roughly assumed to be 3.01 × 10^9^ × 0.13314 = 4.008 × 10^8^, via the proportion of ICC to liver cancer, including ICC (Table S1) during 2015–2019. Although the basis for this estimate is not very firm, it was adopted in our model because a change in the estimated stem cell count does not affect the age-dependent risk estimate (see Fig. S8).

### Mutation frequency of common ICC

Mutation frequencies of occupational and common cholangiocarcinomas have been reported using whole-exome sequencing [12, 13]. The mutation frequencies of four common cases of ICC in two males and two females aged 55–79 were 1.2–1.9 × 10^−6^ (Table S2). These cases suggest that approximately 3000–5000 mutations per genome exist in these cases, and variations among the common carcinomas are small. Approximately 1000–2000 somatic point mutations per genome have been detected in normal human liver stem cells at age 30–55, and the numbers increase with age [27]. Thus, the mutation frequencies of common ICCs are approximately two times higher than those of normal stem cells. As the males and females exhibited similar mutation frequencies, the four common cases were combined. The average mutation frequency was 1.5 × 10^−6^ at an average age of 70.25.

### Incidence and mutation frequencies of occupational ICC

From surgical specimens of four individuals with occupational cholangiocarcinomas, including recurrences, 16 lesions were obtained to analyze their mutation frequencies [12, 13]. Among them, two were extrahepatic lesions, and three were derived from the same clones. Thus, 11 independent intrahepatic clonal lesions existed in the four occupational cases. Characteristics of the four occupational cases and mutation frequencies of 11 independent clones are shown in Table S2. In the case of more than one data from one clone, the highest mutation frequency was adopted. The mutation frequencies of 11 independent clones of occupational ICCs in four patients were significantly higher than those of common ICCs [13]. The high mutation frequencies were caused by occupational exposure to organic solvents. Although six out of 11 were not invasive carcinomas but precancerous lesions, one of them was a subclone whose other subclones were invasive carcinomas, and the remaining five lesions were located within the regions of the biliary intraepithelial neoplasia (BilIN) or intraductal papillary neoplasm of the bile duct (IPNB). Both were regarded as precancerous lesions of cholangiocarcinoma [28] and occupational cholangiocarcinoma [29]. In addition, the average mutation frequencies of precancerous lesions and invasive carcinomas were almost the same, although the frequency of each lesion varied among clones. Therefore, the ICC mutation frequency of the four patients from the 11 clones was estimated. The average mutation frequency was 77.80 × 10^−6^. Except for one patient, the other three patients harbored multiple independent lesions including precancerous lesions caused by independent carcinogenic pathways. Therefore, the exposure conditions, and resulting mutation induction levels estimated in the four patients, would induce multiple independent ICCs in the other persons with the same exposure condition well. As a total of 11 independent lesions were observed in four patients, the average incidence of ICC in the four patients was presumed to be 11/4.

## Results

### Applying the multistage model to occupational and common cholangiocarcinomas

The multistage model [1, 30] demonstrates the probability of cancer incidence after the *k*-th stage in each cell based on the following equation:

1 $${P}_{k}\left(t\right)\approx \frac{1}{k!}{\lambda }_{1}{\lambda }_{2}\cdots {\lambda }_{k}{t}^{k}$$

**Fig. 1:**
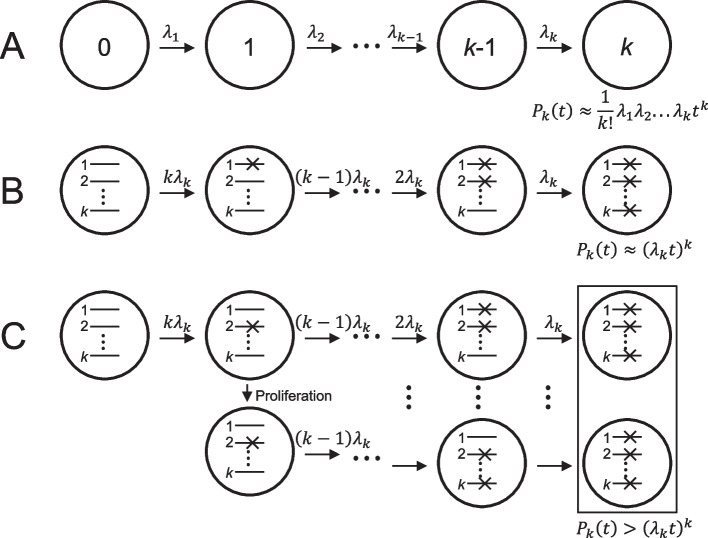
Schematic diagrams of the multistage model with or without mutated cell proliferation. **A**, Armitage–Doll multistage model. The required number of stages for carcinogenesis is *k,* and the event rate for the progression to the next stage is *λ*. The probability equation of the *k*-th stage in each cell is ${P}_{k}\left(t\right)\approx \frac{1}{k!}{\lambda }_{1}{\lambda }_{2}\cdots {\lambda }_{k}{t}^{k}$. **B**, When mutation rates are the same among driver genes, and the number of genes responsible for carcinogenesis is *k*, the probability equation of the *k*-th stage is simplified to ${P}_{k}\left(t\right)\approx {\left({\lambda }_{k}t\right)}^{k}$ (see main text). Although the example in **B** shows that the first mutation occurs in gene 1 and the last mutation occurs in gene *k*, any order of mutations results in malignant transformation. The example in **C** shows different orders of mutations. **C**, Inclusion of mutated cell proliferation. Any proliferated cells become the next stage of acquiring another mutation. Hence, the overall possibility of reaching the next stage will be larger than if no proliferation occurs

For a cell that has experienced an *i*–1 mutational event, the next event rate is *λ*_*i*_ (Fig. 1A). In the model, the cell becomes malignant after acquiring mutations *k* times. Cell expansion in a tissue is not considered. To simplify the calculation, we assume that mutation rates are the same among driver genes and that the number of genes responsible for carcinogenesis is *k*. Hence, the first driver event of carcinogenesis to the next stage occurs when any of the *k* genes acquire a mutation. The second event would occur when any of the *k*–1 genes acquire a mutation. Then, at the *k*-th event, only one gene remains unchanged (Fig. 1B). Hence, the relationship among the rates of each mutational step is given by

2 $${\lambda }_{1}/k={\lambda }_{2}/\left(k-1\right)=\cdots ={\lambda }_{k}/1$$

From Eqs. (1) and (2), the probability of cancer incidence is given by

3 $${P}_{k}\left(t\right)\approx {\left({\lambda }_{k}t\right)}^{k}$$

The target number of mutation sites for the phenotypic change is denoted as *a*; hence, *λ*_*k*_ can be expressed as *a* × *m*, where *m* is a mutation rate per year. Then, the Eq. (3) is converted to

4 $${P}_{k}\left(t\right)\approx {\left({\lambda }_{k}t\right)}^{k}={\left(a\times m\times t\right)}^{k}$$

In this equation, *m* × *t* refers to the accumulated mutation level, *i.e.*, mutation frequency. Among four common ICCs, the average mutation frequency and age were 1.5 × 10^−6^ and 70.25, respectively (Table S2). Therefore, the value *m* was estimated to be 1.5 × 10^−6^/70.25 = 2.135 × 10^−8^. We applied the values to the Eq. (4):

5 $${{P}_{{k}_{(common)}}(70.25)\approx \left(a\times 2.135\times {10}^{-8}\times 70.25\right)}^{{k}_{(common)}}$$

*P*_*k*(*common*)_ and *k*_(*common*)_ indicate the probability of each cell reaching carcinoma and the number of stages in carcinogenesis, respectively. We assume that the carcinogenic mechanism of occupational ICC is the same as that of common ICC, but additional mutational events happen because of occupational exposure to organic solvents. Therefore, the target number of genetic mutations in occupational ICC is the same as that in common ICC, but additional mutational events are calculated during occupational exposure. The additive mutation rate by the exposure and its duration are denoted as *μ* and *τ*, respectively. Then, Eq. (4) is rewritten as

6 $${P}_{k}\left(t,\tau \right)\approx {\left\{a\times \left(\left(m\times t\right)+\left(\mu \times \tau \right)\right)\right\}}^{k}$$

Here, *m* × *t* + *μ* × *τ* represents the mutation frequency of occupational ICC. From the occupational ICC data, the average age of incidence, exposure period, and mutation frequency were 37.1 years, 7.64 years, and 77.80 × 10^−6^, respectively (Table S2). Thus, Eq. (6) for occupational ICCs was given by

7 $${P}_{{k}_{(occupational)}}\left(\text{37.1,7.64}\right)\approx {\left(a \times 77.8\times {10}^{-6}\right)}^{{k}_{\left(occupational\right)}}$$

*k*_(*occupational*)_ represents the number of stages for carcinogenesis in occupational ICC.

As cell proliferation is not considered in the multistage model, malignant transformation probability increases with the acceleration of proliferation during carcinogenesis to a greater extent than that estimated with the multistage model (Fig. 1C). Hence, proliferation partially compensates for the stage numbers of carcinogenesis in the multistage model. The relationship between *k*_(*common*)_ and *k*_(*occupational*)_, which indicates the difference in the contribution of cell expansion, is described in the supplementary material.

### Including the cell expansion effect on the multistage model

In the multistage model, including cell expansion, the coefficients are *λ* and the expansion levels. In this study, we assumed the expansion level to be constant at any stage of progression and denoted it by *s* (expansion/year). For simplification, we assumed the number *k* discretely. Although estimating the total number of cells during expansion was difficult, we assumed that the number of cells in this particular premalignant organ increased with expansion, and the unmutated cells remained. Thus, the number of cells reaching the *i*-th stage after expansion was calculated numerically during a one-year interval as the following difference equation:

8 $${N}_{0}\left(t\right)\approx 4.008\times {10}^{8}$$

9 $$\begin{array}{cc}N_i\left(0\right)=0&\ \left(i > 0\right)\end{array}$$

10 $$N_i\left(t\right)\approx N_i\left(t-1\right)\times\left(s\times i+1\right)+\frac{N_{i-1}\left(t-1\right)+N_{i-1}\left(t\right)}2\times\lambda_i\times\left(\frac{s\times i}2+1\right)\quad \left(i < k\right)\,$$

wherein the first term is a one-year cell expansion that has already reached the *i*-th stage at the beginning of the year. The second term is the number of cells that newly reached the *i*-th stage in one year, which is approximated by the product of the annual average number of the cells that have reached the *i*–1-th stage, event rate for progression to the *i*-th stage (*λ*_*i*_), and expansion of the newly-reached cells during the immediate year. Cells that reached the *i*-th stage continued to appear almost constantly over a year; hence, the average duration of cell expansion during this particular year was approximately one-half a year. Therefore, the expansion contribution was approximated as (s × i)/2.

As the number to be estimated was that of carcinogenic clones rather than cells, the calculation of expansion was unnecessary in the final carcinogenic step. Hence, Eq. (10) is simplified when *i* = *k* and given by

11 $${N}_{k}\left(t\right)\approx {N}_{k}\left(t-1\right)+\frac{{N}_{k-1}\left(t-1\right)+{N}_{k-1}\left(t\right)}{2}\times {\lambda }_{k}$$

**Fig. 2:**
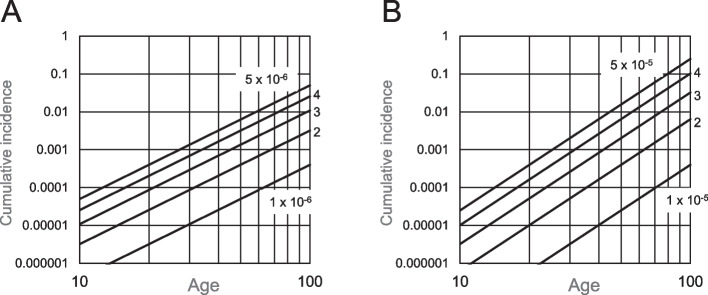
Age-dependent cumulative incidence in the multistage model without cell expansion. Values are calculated from Eq. (3) and assumed number of stem cells responsible for ICC. **A**, *k* = 3; **B**, *k* = 4. Numbers in figures indicate the event rate level *λ*_*k*_

**Fig. 3:**
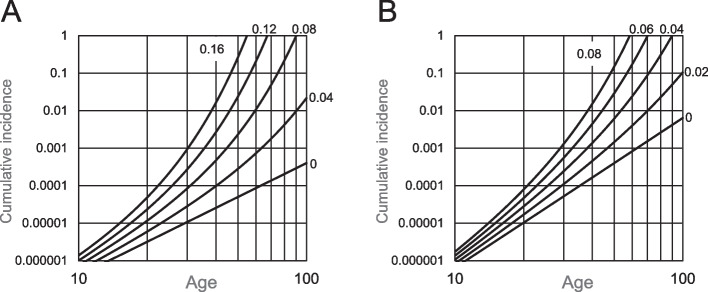
Age-dependent cumulative incidence in the multistage model, including cell expansion. Values are numerically calculated from Eqs. (8), (9), (10), and (11). **A**, *k* = 3 and *λ*_*k*_ = 1 × 10^−6^; **B**, *k* = 4 and *λ*_*k*_ = 2 × 10^−5^. Numbers in the figures indicate cell expansion level *s*

When cell expansion did not occur, the cumulative incidence was calculated to increase linearly with age when the age-incidence relationship was demonstrated under the log–log graph; the slope is the requirement of the number of stages for carcinogenesis, *k*. Figure 2A and B are the examples of *k* being 3 and 4. When the cell expansion effect ***s*** was included in the multistage model, the cumulative incidence was calculated to increase more with aging as per the expansion level (Fig. 3). Therefore, the exponent value of the cumulative incidence vs. age was over *k* and increased with age.

### Simulation of common ICC incidence

**Fig. 4:**
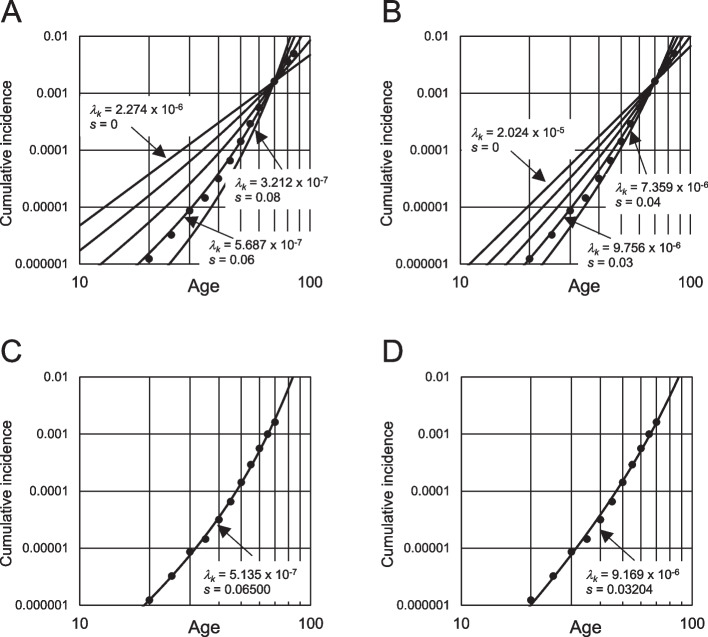
Adjusting event rates and cell expansion levels on the model to common ICC incidence. **A** and **B**, Relationship between *λ*_*k*_ and *s* on age-dependent cumulative incidence. Lines represent changes in cumulative incidence by age in the model under the cumulative incidence at age 70, which is fixed at 0.001617. Expansion level *s* was changed at the same interval, and event rate *λ*_*k*_ was adjusted accordingly. **A**, *k* = 3; **B**, *k* = 4. Circles are the estimated cumulative incidence from the mortality data obtained from Vital Statistics of Japan. **C** and **D**, Regression lines of the model-simulated cumulative incidence to that estimated from the mortality rate. To calculate *λ*_*k*_ and *s* under the non-linear least-squares method, the logarithmic transformed cumulative incidence until age 70 (filled circles) was used. **C**, *k* = 3; **D**, *k* = 4. ICC, intrahepatic cholangiocarcinomas

To simulate the data from Vital Statistics, the mutational event rate *λ*_*k*_ and expansion level *s* were adjusted to match the cumulative incidence of 70 years, estimated from the mortality data in Statistics (Fig. 4A and B). In these figures, the filled circles represent the estimated cumulative incidence based on the mortality rate during 2015–19. For three-stage carcinogenesis (*k* = 3), when *s* was fixed at 0.06, the cumulative incidence was almost that estimated from the mortality. For *k* = 4, *s* = 0.03 demonstrated a similar result. However, the model simulated incidence was higher than that in the older adults when k = 3 and 4; the model had a discrepancy.

Next, *λ*_*k*_ and *s* were calculated from the estimated cumulative incidence using the non-linear least-squares method (NLLSM) after the logarithmic transformation of the incidence. Figure 4C and D demonstrate the regression lines. As the incidence between the model-simulated and estimated values from the mortality data have a discrepancy for extremely old adults, the incidence at age 75 and older was not included in the calculation. Under these conditions, *λ*_*k*_ and *s* were 5.135 × 10^−7^ and 0.06500 (*k* = 3) and *λ*_*k*_ = 9.169 × 10^−6^ and 0.03204 (*k* = 4)*.* The regression lines in *k* = 3 and 4 simulated the cumulative incidence estimated from the mortality well, although the residual sum of squares was lower in the case of *k* = 3.

### Estimating the necessary number of stages for ICC carcinogenesis and the contribution of expansion

The mutation frequency and the incidence of occupational ICC were then included to estimate *k* in the model. The mutation frequency of occupational ICC was expressed as *m* × *t* + *μ* × *τ*. Here, the average age of the 11 occupational ICC cases was 37.1, and the average mutation frequency was 77.80 × 10^−6^. The mutation rate per year, *m*, was estimated as 2.135 × 10^−8^. Therefore, the average mutation level caused by occupational exposure in the ICC cases is

12 $$m\times t+\mu \times \tau =2.135\times {10}^{-8}\times 37.1+\mu \times \tau =77.80\times {10}^{-6}$$

13 $$\mu \times \tau =77.80\times {10}^{-6}-2.135\times {10}^{-8}\times 37.1=77.01\times {10}^{-6}$$

14 $$\frac{\mu \times \tau }{m}=\frac{77.01\times {10}^{-6}}{2.135\times {10}^{-8}}=\text{3,607}$$

Thus, the occupational mutation level was equivalent to that accumulated in 3,607 years without exposure.

For occupational cholangiocarcinomas, patients were diagnosed several years after exposure to causative chemicals until several years after stopping the exposure. The outcome of possible fatal events was up to a few years following diagnosis [11]. Transformation due to exposure would be mostly detected (11 independent clones among four patients) because clones other than the particular cancer that initiated the surgery were identified at the tissue level at the time of surgery. We assumed the preclinical “lag time” between the malignant transformation and surgical resection to be approximately three years. Therefore, the occupational incidence was assumed to reach 11/4 at age 34 years in the model, about three years before the average age, 37.1, at surgery. From the average exposure period of 7.64 years, the exposure period was assumed to be eight years at a constant rate for numerical calculation over a one-year interval.

**Fig. 5:**
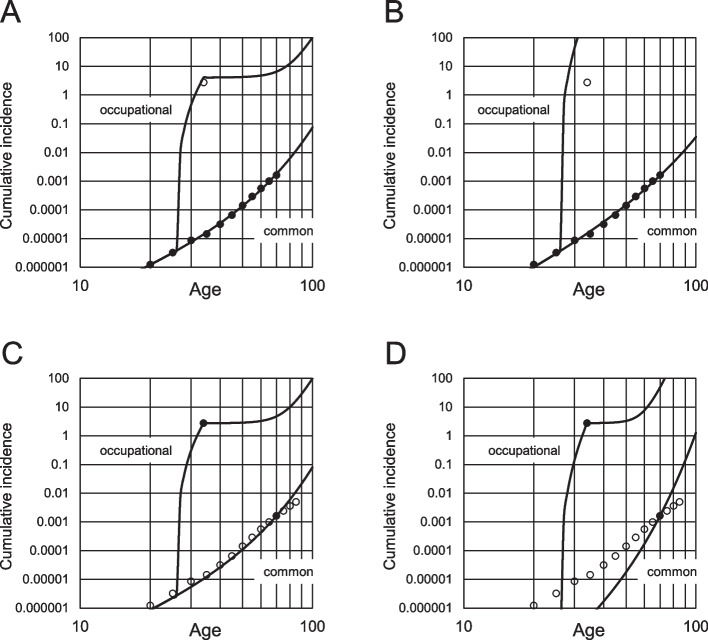
Optimized model simulation according to the estimated incidence of occupational and common ICC and the estimated stages of ICC carcinogenesis. The estimated cumulative incidence at age 70 years in common ICC was 0.001617. The incidence of occupational ICC at the end of exposure was 11/4 = 2.75, which is expressed as an isolated open or filled circle. Circles lined at the bottom of the figures are the estimated cumulative incidence from mortality data obtained from Vital Statistics of Japan. Average mutation frequencies and ages of common and occupational ICC were 1.5 × 10^−6^ at age 70.25 and 77.80 × 10^−6^ at age 37.1, respectively. Occupational mutation level was equivalent to that accumulated in 3,607 years of the ordinary period without exposure (see text). The period and event rate during exposure were set to be the age of 26–34 years and *λ*_*k*_ × (3,607/8 + 1). **A** and **B**, *λ*_*k*_ and *s* were calculated using the non-linear least-squares method from the logarithmic transformed cumulative incidence of the estimated common ICC until age 70 (filled circles). **A**, *k* = 3; *λ*_*k*_ = 5.135 × 10^−7^; *s* = 0.06500. **B**, *k* = 4; *λ*_*k*_ = 9.169 × 10^−6^; *s* = 0.03204. **C** and **D**, *λ*_*k*_ and *s* were adjusted to match the estimated cumulative incidence of common ICC at age 70 and the occupational ICC (filled circles). **C**, *k* = 3; *λ*_*k*_ = 4.460 × 10^−7^; *s* = 0.06865. **D**, *k* = 4; *λ*_*k*_ = 1.985 × 10^−6^; *s* = 0.08237. ICC, intrahepatic cholangiocarcinomas

Figure 5A (*k* = 3) and B (*k* = 4) are the simulated incidence curves of occupational and common ICCs based on NLLSM from the common incidence, the same as those described in Fig. 4C and D. When assuming *k* = 3, the occupational exposure-dependent increase of the model-simulated ICC incidence was almost that of the occupational incidence. For *k* = 4, it was higher than the incidence. Based on this model, these results demonstrated that the number of stages to carcinogenesis, *k*, was approximately three.

When both *s* and *λ* were adjusted to match the estimated cumulative incidence at age 70 as common and occupational ICC incidence rather than using NLLSM from the common incidence, an almost similar model-simulated incidence curve was demonstrated when *k* = 3 (Fig. 5C). In that case, *λ*_*k*_ = 4.460 × 10^−7^ and *s* = 0.06865 were estimated. However, for *k* = 4, the simulated incidence curve and incidence estimated from the mortality of common ICC had a discrepancy (Fig. 5D).

From the value *λ*_*k*_, *k*_(*common*)_ can be calculated from Eq. (3) and (15) (described in the supplementary material), and *k*_(*occupational*)_ can be calculated from Eq. (20). The presumed common and occupational incidences, 0.001617 at age 70 and 11/4, have been used to result in Eq. (20) (see supplementary material). Therefore, the value *λ*_*k*_ = 4.460 × 10^−7^ for *k* = 3, estimated from these cumulative incidences, must be used for the calculation. *k*_(*common*)_ and *k*_(*occupational*)_ were calculated to be 2.529 and 2.927 (Fig. S2 in the supplementary material, the filled square), and the difference in values for *k* = 3 was the cell expansion effect. The value for common ICC was 3 – 2.529 = 0.471, whereas the value for occupational ICC was 0.073. This relatively small value indicates that occupational carcinogenesis occurs without the cell expansion effect at young ages because of the rapid onset of cancer after extensive occupational exposure.

### Occupational exposure and cancer incidence prediction

Under the model conditions, the cumulative incidence of occupational ICC increases rapidly upon exposure. As the mutation level during exposure was much higher than that in the non-exposure period (3607/8 + 1 = 451.9 times each year in the model), occupational mutations were the primary cause of cancer during the exposure period. Therefore, the cumulative incidence was approximated to be proportional to the third power of the exposure period (Fig. S3A). When occupational mutation levels reflecting exposure intensity were changed in the model, the cumulative incidence was also close to the third power of the mutation level (Fig. S3B).

After the exposure, the increase in cumulative risk appeared to stop on the logarithmic graph (Fig. 5). However, the graph by age demonstrated that the age-specific incidence rate of the occupation workers continued to be much larger than that of ordinary individuals (Fig. S4). In addition, the cumulative incidence on the logarithmic graph also shows that it increases again from around age 60 and then rapidly increases with age (Fig. 5A and C). Hence, this model shows that occupational ICCs in those under exposure initially occur during the exposures, which are designated as the immediate risk, and occur again with age, which is the future risk. The clinical lag time often leads to the disease being diagnosed at a late stage and after several years of exposure.

**Fig. 6:**
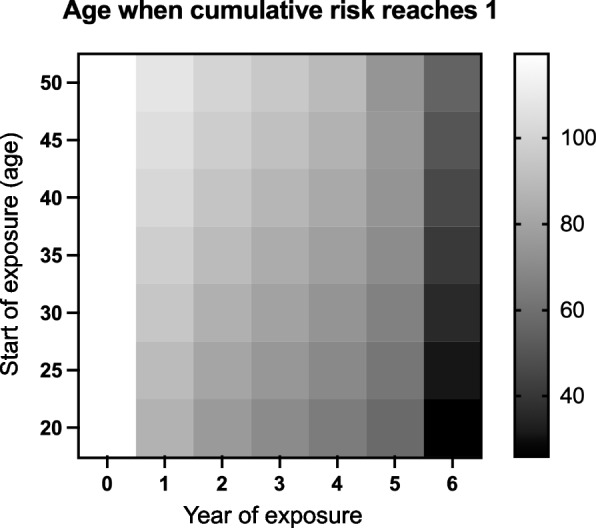
Effect of exposure ages and period on cumulative incidence elevation. The cumulative incidence value of 1 was chosen as a representative of the high risk levels caused by exposure. The event rate during the occupational exposure was *λ*_*k*_ × (3,607/8 + 1). *k* = 3; *λ*_*k*_ = 4.460 × 10^−7^; *s* = 0.06865. The conditions were the same as those described previously (Fig. 5C), except for the starting age of exposure and exposure period

Six-year constant exposure, instead of eight years at the same rate per year, would result in an incidence of 1 (Fig. S3A), indicating that, on average, one case of cancer per person would appear immediately after six years of exposure aged 26 to 32 years. As the incidence would be affected by the ages of the individuals exposed, the exposure period (or amount of exposure), and the post-exposure period, the age when the cumulative incidence reaches one under different ages and periods, were estimated (Fig. 6). Between 1–4 years of exposure at the same rate, an older age of the individuals exposed indicated an older age of reaching the cumulative incidence of 1, at roughly the same extent. This means that such an extensive risk will appear after a certain period following the end of exposure, regardless of the exposure age. In addition, five years of exposure at any age will not reach an immediate risk value of 1. Therefore, the necessary levels of exposure to reach the risk of 1 is approximately six years, regardless of the age of the individuals exposed.

**Fig. 7:**
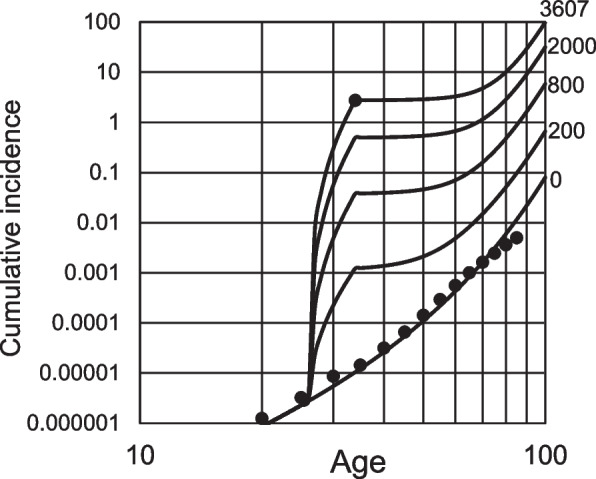
Estimation of the ICC cumulative incidence risk under the different mutation induction levels caused by occupational exposure. *k* = 3; *λ*_*k*_ = 4.460 × 10^−7^; *s* = 0.06865. The exposure period was set to be the age of 26–34. Numbers in the figures indicate mutation induced by exposure corresponding to years without exposure. ICC, intrahepatic cholangiocarcinomas

This model demonstrated the occupational risk in this study based on the actual incidence and mutation frequency of the four patients with multiple cancers. However, the risk of occupational cancers, including ICC, was lower than in the cases mentioned in the previous section [31]. Hence, the estimated exposure levels of most workers were expected to be lower than the case in this study. To evaluate the immediate and future risks of workers with different levels of exposure, various occupational mutation levels were applied to the model, and the cumulative incidence was demonstrated (Fig. 7). As shown in Fig. S3, the immediate risk was almost proportional in the third power to the mutational burden; hence, changing levels largely affects the incidence. Thus, each worker with different exposure levels would have a significantly different immediate risk level. In addition, the future risk among the workers with different exposure levels was also different. However, the future risk levels were relatively higher than the immediate risk levels in workers exposed to lower levels (Fig. S5) under the relative contributions of common mutations and intermediate cell expansion. Therefore, an individual risk with a low exposure level will be lower than that with a high exposure level, even in the future; however, the future risk should concern the population with a low exposure level.

In this model analysis, many parameters were presumed. Hence, we also examined the case where *k* = 3, and the other parameters were sequentially changed. The details of the results are provided in the supplementary material.

## Discussion

The number of stages of carcinogenesis in the model directly affects the age-dependent increase in risk estimation. When the reported mutation frequencies and occupational ICC incidences were applied to this model, carcinogenesis was estimated to occur in three stages (Fig. 5A and C). When four stages were assumed, there was a large discrepancy with estimated mutation frequencies or incidences (Fig. 5B and D). At the same time, the ratio of mutation frequencies in common and occupational ICCs significantly affected the slope (Fig. S9). Therefore, accurately estimating the mutation frequencies of both ICCs is essential to estimate the decimal level of the effective number of stages in the model. In this study, mutation frequency was estimated from 11 occupational ICCs and four common ICCs. Therefore, the estimation was not very accurate and should be mentioned as a limitation for estimating the stage number of carcinogenesis in this study. In addition, the incidence at the end of occupational exposure also affected it (Fig. S10), although the effect on the slope is not as high as in the case of the ratio of mutation frequencies. Other parameters presumed in this study showed relatively minor effects on the model estimates. Different assumptions of the number of stem cells responsible for ICC did not affect the slope. Although the slope is affected by the mutation frequency estimation, it is generally demonstrated as a temporary decrease in the risk of cancer after stopping occupational exposure and an increase in the risk of cancer once more in older adults.

The estimated number of ICC stages in this study was less than the number of cancer hallmarks [32]. In contrast, the incidence of colorectal cancer was predicted well using the three-stage clonal expansion model [5]. Comparing the mutation rates and incidences of high-risk and control groups indicates that three driver gene mutations are necessary for lung and colorectal cancer development [33]. Furthermore, 7,664 tumors across 29 cancer types carry an average of four coding substitutions under positive selection determined from the dN/dS ratio [34]. The average number of stages for hepatocellular carcinoma is also reported to be approximately four. Although the model-estimated number in this study was three, further driver mutations may be acquired during the clinical progression of the cancers. To consider the second round of clonal expansion after intermediate transformation on the multistage clonal expansion model, approximation of the second clonal expansion as the existence of clinical lag time (MSCE-1 approximation) enables the estimation of various cancers [35]. Altogether, the estimated number of stages necessary for carcinogenesis in this study is consistent with that of previous studies.

The model in this study indicated that high somatic mutation levels induced by occupational exposure resulted in the ICC and proliferation effects being small. This case would be similar to certain cases of hereditary cancers, such as early-onset colorectal cancers with a high tumor mutation burden caused by pol ε or mismatch repair gene mutations [36, 37]. Although high mutation induction levels owing to occupational exposure cause occupational cholangiocarcinomas, the resultant high tumor mutation burden of the cancers suggests that immunotherapy may be effective [38]. In addition, the occupational tumors showed the expression of programmed cell death ligand 1 in cancer cells and the presence of programmed cell death 1-positive T cells and CD-8 positive lymphocytes [39]. The treatment and efficacy of nivolumab in patients with occupational cancer have been reported [40].

As mentioned, the model in this study predicted the cumulative incidence estimated from the mortality of ICC until the age of 60. However, in older adults, a clear divergence was observed (Figs. 4 and 5). This may represent a limitation of the relatively simplified model used in this study. The model uniformly defined the cell expansion rate; hence, the exponent value of the cumulative incidence versus age increased with age in the model. However, the value estimated from the mortality data decreased at age 60 and above (Fig. S12), resulting in divergence.

The increment of cancer incidence rate slowing in older adults has been discussed. For example, cell senescence with aging [41] and lineage disappearance during somatic evolution under clonal expansion [42] are proposed as models. Somatic evolution under the linear process rather than logarithmic function [43], compartment size-dependent mutant accumulation [44], and cellular interaction [45] were also proposed. Malignant transformation often occurs from a premalignant tumor, such as a polyp that presents for a long time without noticeable growth. Such a transformation indicates that premalignant cells do not always grow logarithmically. In some cases of chronic lymphocytic leukemia, cells demonstrate logistic growth and reach a certain steady-state level [46]. In addition, many mutated clones that are phenotypically normal expand in various normal tissues [47, 48]. Such expansions simultaneously occur in the shared tissue area, suppress clonal growth, and cause lineages to disappear. Apart from these considerations, decreasing diagnostic intensity may also contribute to a decline in cancer incidence among older adults [49]. It will be important to include recent progress in genome research in cancer model analysis to assess the risk of occupational ICC more accurately [50–52].

Cancer incidence in this model depended solely on mutations, presumed to occur via time-and-exposure-dependent and time-dependent expansion. The development of new cancers almost always stops via stopping exposure in the model (Fig. 5 and Fig. S4). After three years of the transformation, clinical cancer may appear in the model. Thus, the clinical risk of occupational cancer was predicted to drop sharply after three years following the end of occupational exposure. However, the clinical lag time is unlikely to be uniformly three years. In addition, continuous mutational events may occur even after exposure, caused by the damage and inflammation of the tissue or the remaining DNA adducts, etc. Hence, overt occupational cholangiocarcinoma may occur in the future, even after exposure is withdrawn. The cases have already been reported approximately ten years after the end of exposure [9].

For the exposed individuals, the risk of cancer temporally decreased, but it is still much higher than in unexposed individuals (Fig. S4). In addition, the risk may rise again as the future risk, caused by the expansion. Cases of cholangiocarcinoma found at the ages of 18 and 22 years after the end of exposure have been reported recently [53, 54]. The classification of cases as “immediate risk” with unusually long clinical lag time or as “future risk” caused by the expansion is unknown. The incidence of new cases in the next decade may provide validity to the model predictions and estimation of future risk. The model indicates that occupational workers are at a higher risk of cancer as they age. In addition, over-estimation in the older adult population already mentioned is also expected to be true for occupational cholangiocarcinoma. Therefore, even if the model estimates are generally correct, the actual risk of occupational cholangiocarcinoma in older adults is expected to be lower than that predicted by the model. Regardless of the model validity, a higher risk can be expected to exist than in the general population. Therefore, careful follow-up throughout life is essential.

## Conclusions

Mutation frequencies of occupational cholangiocarcinomas among workers in printing are significantly higher than in common cases of cholangiocarcinomas. A multistage model that included the cell expansion phenomenon at all carcinogenesis stages was applied and compared with occupational cases, common cases, and Vital Statistics in Japan. Three-stage ICC carcinogenesis was proposed from the model. Event rate *λ*_*k*_ and expansion *s* for each phenotypic change were estimated to be 4.460 × 10^−7^ and 0.06865 per year. Under this model, the predicted incidence curve was similar to that estimated from the mortality rate of ICC, except that in older adults. The model predicted an increase in the risk of cancer once more in older adults.

## Supplementary Information

Additional file 1: Supplementary Material 1. Supplementary test.

Additional file 2: Supplementary Material 2. Supplementary Tables 1 and 2, Supplementary Figs. 1–12.

## Data Availability

All data in this study have been referred from published sources and appear in this article, including the supplementary material.

## Abbreviations

ICC: Intrahepatic cholangiocarcinoma
BilIN: Biliary intraepithelial neoplasia
IPNB: Intraductal papillary neoplasm of bile duct
NLLSM: Non-linear least-squares method
