# Supplementary material for: Multistage carcinogenesis in occupational cholangiocarcinoma: the impact of clonal expansion and risk estimation
Source: Genes Environ. 2024 Oct 24;46:21. doi: 10.1186/s41021-024-00315-7 (PMC11515581; doi:10.1186/s41021-024-00315-7)
Supplement: Supplementary file 1 — Additional file 1: Supplementary Material 1. Supplementary test. [file 41021_2024_315_MOESM1_ESM.docx]

Supplementary text

**Relationship of apparent multistage model stages between occupational and common intrahepatic cholangiocarcinomas**

Proliferation partially compensates for the carcinogenesis stage number in the multistage model. Therefore, the required “apparent” number of stages in the multistage model to reach the particular probability value is lower than the “actual” number for carcinogenesis.

The cumulative incidence of intrahepatic cholangiocarcinoma (ICC) at age 70, closest to the age 70.25 in Eq. (5), was estimated to be 0.001617 (Table S1). Therefore, the probability of common ICC at age 70 in each cell is calculated to be

(15)

$$P_{k_{(common)}}\left( 70 \right)=\frac{0.001617}{4.008\times{10}^{8}}=4.034\times{10}^{-12}$$

where 4.008 × 10^8^ is the assumed number of stem cells responsible for ICC. The value is applied to Eq. (5), modified with the age, and is given by

$\left( a\times2.135\times{10}^{-8}\times70 \right)^{k_{(common)}}\approx4.034\times{10}^{-12}$ (16)

Thus,

(17)

$$a\approx\frac{1}{70\times2.135\times{10}^{-8}}\times\left( 4.034\times{10}^{-12} \right)^{\frac{1}{k_{\left( common \right)}}}=6.691\times{10}^{5}\times\left( 4.034\times{10}^{-12} \right)^{\frac{1}{k_{\left( common \right)}}}$$

The occupational incidence is presumed to be 11/4 (see Methods). Therefore, the probability in each cell is calculated to be

(18)

$$P_{k_{(occupational)}}\left( 37.1,7.64 \right)=\frac{{11}/4}{4.008\times{10}^{8}}=6.861\times{10}^{-9}$$

The value is applied to Eq. (7) and is given by

$\left( a\times77.80\times{10}^{-6} \right)^{k_{(occupational)}}\approx6.861\times{10}^{-9}$ (19)

From Eq. (17) and (19), the relationship between *k*_(_*_common_*_)_ and *k*_(_*_occupational_*_)_ is given by

$$k_{\left( occupational \right)}\approx\frac{\log\left( 6.861\times{10}^{-9} \right)}{\log\left[ 6.691\times{10}^{5}\times\left( 4.034\times{10}^{-12} \right)^{\frac{1}{k_{\left( common \right)}}}\times77.80\times{10}^{-6} \right]}=\frac{-18.797}{3.9523-\frac{1}{k_{\left( common \right)}}\times26.236}=\frac{0.7165}{\frac{1}{k_{\left( common \right)}}-0.1506}$$

The relationship between *k*_(_*_common_*_)_ and *k*_(_*_occupational_*_)_ is shown in Fig. S2. When *k*_(_*_common_*_)_ was assumed to be 3, *k*_(_*_occupational_*_)_ was 3.921. The difference between these apparent numbers is because of the differences in cell expansion effects; *k*_(_*_common_*_)_ is smaller owing to long-term expansion in common ICC. If *k*_(_*_common_*_)_ was assumed to be 2, *k*_(_*_occupational_*_)_ was 2.051.

(20)

**Effects of various factors on model simulation**

We examined a case where *k* = 3 was fixed, and the other parameters were sequentially changed to elucidate the effect of the age-dependent cumulative incidence curve on the model. If the lag time from the malignant transformation to death in the model was changed to 10 years instead of 5 years, the estimated cumulative incidence from the mortality shifts at relatively young ages based on an extended presumed lag time. This change caused the model simulation slope to be steeper because a higher expansion level was estimated to reach the fixed cumulative incidence at a younger age, from 75 to 65, according to the lag time of 0–10 years. Thus, a considerable divergence appeared between the model simulations and estimates of the mortality rate (Fig. S6). This phenomenon is almost the same regardless of the different assumptions of the occupational ICC lag time, be it together or not.

When the survival rate was assumed to be 0% or 40% instead of 20%, the estimated cumulative incidence increased with the survival rate. Hence, the expansion was adjusted to be high to follow it in the model. This adjustment caused the model to diverge in simulation and estimation from the mortality rate (Fig. S7). However, within the range in this study, the divergence was smaller than the effect of the lag time, as mentioned previously.

Different assumptions of the number of stem cells responsible for ICC did not affect the model simulation except for the event rate per cell, *λ* (Fig. S8). *λ_k_* was inversely proportional to the cube root of the assumed number of stem cells when the stage number was assumed to be three.

The ratio of common and occupational mutation frequencies affected the model. As long as the ratio did not change, their absolute values did not affect the model. When the ratio changed to 103.7 (or 25.93), which is 2- (or 1/2-) fold of the initially estimated ratio, 77.80/1.5 = 51.87, a relatively increased (decreased) event rate during the exposure increased (decreased) the incidence during exposure if *k* was fixed. As the incidence at the end of exposure was fixed, the incidence prior to exposure decreased (increased). Thus, the age-dependent increase was higher (lower) than the original simulations, resulting in a large divergence between the model simulations and the estimate from the mortality rate (Fig. S9).

When the assumed incidence of occupational ICC at the end of the exposure was changed from 11/4 = 2.75 to 1.375 or 5.5, the incidence prior to exposure also changed because the ratio of common and occupational mutation frequencies was fixed. The age-dependent increasing levels on the model also changed accordingly, which caused the divergence between the model simulation and mortality rate estimate, although this effect did not appear extensive (Fig. S10).

The exposure and accumulation of mutation caused a high occupational immediate risk (see Fig. 5). When the exposure period was moved from 26–34 to 5 years before and after without changing the lag time, virtually no change occurred in the model (Fig. S11) except that the exposure period directly affected the ages at which future risks may arise, in line with the simulation described in Fig. 6.
