# Supplementary material for: Multistage carcinogenesis in occupational cholangiocarcinoma: the impact of clonal expansion and risk estimation
Source: Genes Environ. 2024 Oct 24;46:21. doi: 10.1186/s41021-024-00315-7 (PMC11515581; doi:10.1186/s41021-024-00315-7)
Supplement: Supplementary file 2 — Additional file 2: Supplementary Material 2. Supplementary Tables 1 and 2, Supplementary Figs. 1–12. [file 41021_2024_315_MOESM2_ESM.docx]

**Supplementary Table 1. Estimating cumulative incidence of intrahepatic cholangiocarcinoma (ICC) in males.**

Population and death from ICC were referred from Vital Statistics in Japan.

age group total 0–4 5–9 10–14 15–19 20–24 25–29 30–34 35–39 40–44 45–49 50–54 55–59 60–64 65–69 70–74 75–59 80–84 85–89 90–94 95–99 100–

*population (male)*

1999 61358000 3020000 3066000 3439000 3889000 4494000 4944000 4298000 3922000 3918000 4685000 4878000 4346000 3674000 3266000 2565000 1484000 862000 451000 157000

2015 61022756 2528080 2698523 2855328 3073597 3014733 3210180 3652706 4191265 4922423 4365334 3982000 3749854 4181397 4699236 3608735 2806665 2009820 1065311 335740 63468 8361

2016 60866773 2504389 2689161 2798896 3058390 3026264 3126981 3578557 4033393 4854270 4625678 3919357 3724301 3990242 4946701 3435607 2894568 2089763 1117351 377000 67000 9000

2017 60675736 2475347 2659147 2755513 3030148 3038927 3063386 3502750 3907152 4716029 4715606 4047688 3748913 3817694 4773046 3611404 2997375 2150399 1170638 416000 70000 9000

2018 60454898 2439327 2621279 2732573 2979988 3063388 3011184 3407503 3804868 4537753 4820728 4150617 3779120 3714721 4506587 3850818 3089021 2187107 1222397 451000 76000 9000

2019 60208034 2396769 2575221 2711090 2927427 3063070 2999849 3300059 3724188 4341201 4891569 4254560 3809196 3682399 4191592 4075907 3225463 2191157 1270870 481000 87000 9000

*death by ICC (male)*

1999 1075 0 0 0 0 0 1 3 2 15 26 45 86 137 215 207 135 126 56 16 5 0

2015 2175 0 0 0 0 1 2 1 1 18 33 54 100 162 311 378 366 397 245 90 13 2

2016 2139 0 0 0 0 0 1 4 4 7 23 49 87 164 325 347 405 385 237 78 22 1

2017 2243 0 0 0 0 1 0 4 3 13 25 58 84 167 355 360 415 379 259 99 19 2

2018 2180 0 0 0 0 0 0 4 7 12 21 48 85 175 320 342 374 403 272 97 19 1

2019 2328 0 0 0 0 1 2 2 3 14 24 40 93 161 309 410 418 407 289 130 23 2

*ICC mortality rate* (×10^−6^)

1999 0 0 0 0 0 0.2023 0.698 0.5099 3.828 5.55 9.225 19.79 37.29 65.83 80.7 90.97 146.2 124.2 101.9

2015 0 0 0 0 0.3317 0.623 0.2738 0.2386 3.657 7.56 13.56 26.67 38.74 66.18 104.7 130.4 197.5 230 268.1 204.8 239.2

2016 0 0 0 0 0 0.3198 1.118 0.9917 1.442 4.972 12.5 23.36 41.1 65.7 101 139.9 184.2 212.1 206.9 328.4 111.1

2017 0 0 0 0 0.3291 0 1.142 0.7678 2.757 5.302 14.33 22.41 43.74 74.38 99.68 138.5 176.2 221.2 238 271.4 222.2

2018 0 0 0 0 0 0 1.174 1.84 2.644 4.356 11.56 22.49 47.11 71.01 88.81 121.1 184.3 222.5 215.1 250 111.1

2019 0 0 0 0 0.3265 0.6667 0.606 0.8055 3.225 4.906 9.402 24.41 43.72 73.72 100.6 129.6 185.7 227.4 270.3 264.4 222.2

2015–19 average 0 0 0 0 0.1974 0.3219 0.8627 0.9287 2.745 5.419 12.27 23.87 42.88 70.2 98.97 131.9 185.6 222.7 239.7 263.8 181.2

*ICC cumulative mortality* (×10^−6^)

age 5 10 15 20 25 30 35 40 45 50 55 60 65 70 75 80 85 90 95 100

2015–19 average 0 0 0 0 0.9872 2.597 6.91 11.55 25.28 52.37 113.7 233.1 447.5 798.5 1293 1953 2881 3994 5192 6511

*ICC cumulative incidence (presumed)* (×10^−6^)

age 20 25 30 35 40 45 50 55 60 65 70 75 80 85 90 95

2015–19 average 1.234 3.246 8.638 14.44 31.6 65.47 142.2 291.3 559.4 998.1 1617 2441 3601 4993 6490 8139

From number of male births in 1910–14 and 1930–34, which were 4473956 and 5391314, respectively (Vital Statistics in Japan), and population of age group 85–89, age group survival rates in 1999 and 2019 were 10.1% and 23.6%, respectively, although small error occurred wherein population number was estimated until October 1, rather than December 31. Number of deaths from liver cancer and ICC were 22224, 19008, 15992, 17822, 17032, and 14222 in 1999, 2015, 2016, 2017, 2018, and 2019, respectively.

**Supplementary Table 2. Patient characteristics.**

age gender exposure period mutation frequency

*common case*

1 55 male 1.4 × 10^−6^

2 73 male 1.5 × 10^−6^

3 72 female 1.2 × 10^−6^

4 79 female 1.9 × 10^−6^

average^a^ 70.25 1.5 × 10^−6^

*occupational case*

1 40 male 11 years 11 months 110.4 × 10^−6^, 15.4 × 10^−6^

2 39 male 7 years 4 months ^b^113.6 × 10^−6^, ^b^6.9 × 10^−6^, ^b^85.9 × 10^−6^, ^b^146.3 × 10^−6^

3 31 male 6 years 6 months 53.9 × 10^−6^

4 34 male 6 years 1 month 85.6 × 10^−6^, ^b^89.5 × 10^−6^, ^b^47 × 10^−6^, 101.3 × 10^−6^

average^a^ 37.1 7.64 years 77.80 × 10^−6^

All data in this table have been referred from Mimaki et al. (*Carcinogenesis*, 2016, 37, 817) and Mimaki et al. (*Carcinogenesis*, 2020, 41, 368).

^a^ average of four common or 11 occupational samples. 0.5 years was added to the data to compensate for an elapsed period following birthdays

^b^ precancerous lesion

**Supplementary Fig. 1. Mortality rates of male ICC by age in 1999 and 2019**

**Supplementary Fig. 2. Relationship between common and occupational ICC on apparent numbers of *k*, stage number for carcinogenesis in the ordinary multistage model without cell expansion**

Cell expansion elevates the incident risk; hence, the apparent number *k* is smaller than the actual *k*. The difference between the apparent and actual *k* is higher in common ICCs, as most of these are diagnosed in relatively older adults. Filled circles indicate two examples of the case (see supplementary text). Filled square is the estimation from the multistage modified by including the cell expansion model in this study.

**Supplementary Fig. 3. Exposure and risk of ICC during exposure in the model**

Thin lines indicate cubic tangent lines on the log-log scale. A, Time-dependent risk. Event rate during the exposure period was (3,607/8 + 1) times the event rate without exposure, *λ_k_*. Other conditions, including the start of exposure at age 26 years, were the same as those demonstrated in Figure 5C; *k* = 3; *λ_k_* = 4.460 × 10^−7^; *s* = 0.06865. Eight years of exposure leads to an incidence of 2.75. B, Exposure-intensity-dependent risk. Numbers in x-axes of figures indicate mutations induced via exposure corresponding to years without exposure. Other conditions, including exposure period at age 26–34 years, were the same as those demonstrated in Fig. 5C.

**Supplementary Fig. 4. Estimated incidence rate on optimized model according to estimated incidence of occupational and common ICC**

The estimated cumulative incidence at age 70 for common ICC was 0.001617, and the incidence of occupational ICC at the end of exposure was 11/4 = 2.75. The period and event rate during exposure were set to age 26–34 years and *λ_k_* × (3,607/8 + 1). *λ_k_* and *s* were adjusted to the incidence. *k* = 3; *λ_k_* = 4.460 × 10^−7^; *s* = 0.06865. Filled circles lined at the bottom of figure are the estimated incidence rates from the mortality data obtained from the Vital Statistics of Japan.

**Supplementary Fig. 5. Immediate and future risk based on different exposure levels**

Numbers in graph area indicate the ratio of the contribution of future risk until age 70 to the immediate risk.

**Supplementary Fig. 6. Cumulative incidence curve changes on different assumptions of lag time from malignant transformation to fatal event**

Different assumptions were adopted on common cases alone (A) or both common and occupational cases (B). Model-estimated cumulative incidences are expressed as lines in figures. *λ_k_* and *s* were adjusted to match the estimated cumulative incidence from cumulative mortality at age 75 years and the occupational incidence, both shown by filled marking points. In both common and occupational cases, lower right lines and squares, center lines and circles, and upper left lines and triangles indicate 0, 5, and 10 years of the lag times, respectively, in figures A and B. Marking points lined at the bottom of figures are the estimated cumulative incidence from the mortality data obtained from Vital Statistics of Japan. Each point on the occupational simulation lines indicates occupational incidence at the end of exposure, 11/4 = 2.75.

**Supplementary Fig. 7. Cumulative incidence curve changes on different assumptions of ICC survival rates**

Cumulative incidences of model estimates are expressed as lines in figures. *λ_k_* and *s* were adjusted to match the estimated cumulative incidence at age 70 years and occupational incidence, indicated by filled marking points. In both common and occupational cases, lower right lines and squares, center lines and circles, and upper left lines and triangles indicate 0, 20, and 40% survival rates, respectively. Marking points lined at the bottom of figures are the estimated cumulative incidence from mortality data obtained from Vital Statistics of Japan. Filled circle on the estimated occupational lines indicates occupational incidence at the end of exposure, 11/4 = 2.75.

**Supplementary Fig. 8. Cumulative incidence curve changes on different assumptions of the number of stem cells responsible for ICC**

*λ_k_* and *s* were adjusted to match the estimated cumulative incidence at age 70 years and occupational incidence, indicated by filled marking points. In both common and occupational cases, changes were not observed in the cumulative incidence curves by the change in the number of stem cells from 2.004 × 10^8^, 4.008 × 10^8^, and 8.016 × 10^8^. Therefore, only a curve in both cases appeared in this figure. The event rate per cell, *λ_k_*, was calculated to be 5.619 × 10^−7^, 4.460 × 10^−7^, and 3.540 × 10^−7^, respectively.

**Supplementary Fig. 9. Cumulative incidence curve changes on different assumptions of the ratio of common and occupational mutation frequencies**

*λ_k_* and *s* were adjusted to match the estimated cumulative incidence at age 70 years and occupational incidence, indicated by filled marking points. In both common and occupational cases, looser, center, and steeper lines indicate 1/2, 1, and 2 times the original estimation ratio (77.80/1.5 = 51.87), respectively. Circles lined at the bottom of figures are the estimated cumulative incidences from mortality data obtained from Vital Statistics of Japan. Filled circle on the estimated occupational lines indicates occupational incidence at the end of exposure, 11/4 = 2.75.

**Supplementary Fig. 10. Cumulative incidence curve changes on different assumptions of incidence at the end of occupational exposure**

*λ_k_* and *s* were adjusted to match the estimated cumulative incidence at age 70 years and occupational incidence, indicated by filled marking points. In both common and occupational cases, lower right, center, and upper left lines indicate (11/4)/2 = 1.375, 11/4 = 2.75, and 11/4 × 2 = 5.5, respectively. Each point on the occupational simulation lines indicates occupational incidences at the end of exposure. A filled square, circle, and triangle indicate incidence assumption to be 1.375, 2.75, and 5.5, respectively. Circles lined at the bottom of figures are the estimated cumulative incidences from mortality data obtained from Vital Statistics of Japan.

**Supplementary Fig. 11. Cumulative incidence curve changes on different assumptions of exposure ages**

*λ_k_* and *s* were adjusted to match the estimated cumulative incidence at age 70 years and occupational incidence, indicated by filled marking points. In occupational cases, the right, center, and left lines indicated exposure periods between ages 31–39, 26–34, and 21–29, respectively. Each point on the occupational simulation lines indicates occupational incidence at the end of exposure, 11/4 = 2.75. A filled square, circle, and triangle represent exposure at 31–39, 26–34, and 21–29, respectively. Circles lined at the bottom of figures are the estimated cumulative incidences from mortality data obtained from Vital Statistics of Japan.

**Supplementary Fig. 12. Value of exponents of age-dependent cumulative incidence vs. age.**

Dashed line demonstrates a monotonous increase from approximately 4 at the age of 20 to 10 at the age of approximately 75, which is the calculated exponent value of the model. Solid line is the estimated value from the mortality data obtained from Vital Statistics of Japan.
